# Altered pupillary light responses are associated with the severity of autonomic symptoms in patients with Fabry disease

**DOI:** 10.1038/s41598-021-87589-x

**Published:** 2021-04-14

**Authors:** Gulfidan Bitirgen, Kultigin Turkmen, Nazmi Zengin, Rayaz A. Malik

**Affiliations:** 1 Department of Ophthalmology, Necmettin Erbakan University Meram Faculty of Medicine, Konya, Turkey; 2 Division of Nephrology, Department of Internal Medicine, Necmettin Erbakan University Meram Faculty of Medicine, Konya, Turkey; 3 Weill Cornell Medicine-Qatar, Research Division, Qatar Foundation, Doha, Qatar; 4 Institute of Cardiovascular Sciences, Cardiac Centre, Faculty of Medical and Human Sciences, University of Manchester, Manchester, UK

**Keywords:** Eye manifestations, Neurological manifestations, Peripheral neuropathies

## Abstract

Symptoms of autonomic dysfunction are common in Fabry disease. In this study we aimed to evaluate alterations in the pupillary response to white light stimulation in patients with Fabry disease and their association with the severity of autonomic symptoms. Fourteen consecutive patients with Fabry disease and 14 healthy control participants were enrolled in this cross-sectional study. The Mainz Severity Score Index (MSSI) was used to measure the severity of Fabry disease and the Composite Autonomic Symptom Scale 31 (COMPASS 31) questionnaire was used to evaluate the severity of autonomic symptoms. The pupil light responses were assessed with an infrared dynamic pupillometry unit. There were significant reductions in the amplitude (*P* = 0.048) and duration (*P* = 0.048) of pupil contraction, and the latency of pupil dilation (*P* = 0.048) in patients with Fabry disease compared to control subjects. The total weighted COMPASS 31 score correlated with MSSI (*r* = 0.592; *P* = 0.026) and the duration of pupil dilation (ρ = 0.561; *P* = 0.037). The pupillomotor weighted sub-score of the COMPASS 31 correlated inversely with the duration of pupil contraction (*r* = − 0.600; *P* = 0.023) and latency of pupil dilation (ρ = − 0.541; *P* = 0.046), and directly with the duration of pupil dilation (ρ = 0.877; *P* < 0.001) and MSSI (*r* = 0.533; *P* = 0.049). In conclusion, abnormal pupillary function is demonstrated in patients with Fabry disease, which is associated with the severity of autonomic symptoms.

## Introduction

Fabry disease is an X-linked lysosomal storage disorder caused by mutations in the gene encoding the enzyme α-galactosidase A, leading to the accumulation of glycosphingolipids, in particular globotriaosylceramide in various tissues including kidney, myocardium, blood vessels, cornea, skin, and neural tissues^1,2^. A major symptom of Fabry disease is neuropathic pain, attributed to glycosphingolipid accumulation in the dorsal root ganglia and associated small fiber neuropathy^3,4^. Accumulation of glycosphingolipid in the central autonomic nuclei and peripheral autonomic nerves has also been demonstrated^4–6^ and a skin biopsy study has shown axonal degeneration in peripheral autonomic nerve fibers^7^. The manifestations of autonomic dysfunction in Fabry disease include impaired sweating, reduced saliva and tear production, altered gastrointestinal motility, arrythmias and orthostatic intolerance^1,8–10^. Detailed studies evaluating the sympathetic skin response, quantitative sudomotor axon reflex test and heart rate variability to forced breathing have confirmed autonomic dysfunction in Fabry disease^1,10–12^.

Dynamic pupillometry is a rapid, non-invasive screening method to quantify autonomic dysfunction and requires minimal specialist training^13,14^. It allows quantitative measurement of pupillary responses to a light stimulus and provides a measure of autonomic innervation of the iris. Pupillomotor function has been found to be altered in Alzheimer’s disease, Parkinson’s disease, multiple sclerosis, diabetes, and overactive bladder^14–18^. The aim of this study was to evaluate the pupillary light responses in patients with Fabry disease in relation to disease severity and the severity of autonomic symptoms.

## Results

The main characteristics of the patients with Fabry disease and control participants are given in Table 1. There were no significant differences between patients and controls for age (*P* = 0.982) and gender (*P* > 0.999). The patients with Fabry disease were members of 3 different families and the detected gene mutations were c.100A > C (p.N34H) in 10 patients, c.160C > T (p.Leu54Phe) in 2 patients, and c.1072_1074delGAG (p.358delE) in 2 patients. The mean value of the α-galactosidase A enzyme activity was 1.41 ± 0.78 µmol/L/h. Eleven patients (79%) were on enzyme replacement therapy (ERT) (biweekly infusions of 0.2 mg/kg agalsidase alpha; Replagal, Shire Human Genetic Therapies AB, Sweden), and the mean duration of ERT use was 24.2 ± 10.8 months. Three patients (2 females; aged 52 and 61 years, and 1 male; aged 42 years) were not receiving ERT and the scores of MSSI were 13 and 20 in female patients and 36 in the male patient. The females were not receiving ERT as they had only recently been diagnosed and the male refused treatment. The mean value of the MSSI was 20.7 ± 8.7 and the mean value of the COMPASS 31 total weighted score was 28.5 ± 14.5.

**Table 1:** Baseline characteristics.

	Controls (n = 14)	Fabry disease (n = 14)
Age (years, median [IQR])	30.5 [26.8–40.5]	30.0 [27.3–42.0]
Sex (F/M)	6/8	6/8
α-Galactosidase A enzyme activity (µmol/L/h)	–	1.41 ± 0.78
Duration of ERT (months)	–	24.2 ± 10.8
MSSI	–	20.7 ± 8.7
COMPASS 31 Total weighted score	–	28.5 ± 14.5
COMPASS 31 Pupillomotor weighted sub-score	–	2.2 ± 0.9

Data are expressed as mean ± SD for parametric variables and median [IQR] for non-parametric variables.

*COMPASS* Composite Autonomic Symptom Scale, *ERT* enzyme replacement therapy, *MSSI* Mainz Severity Score Index.

**Figure 1:**
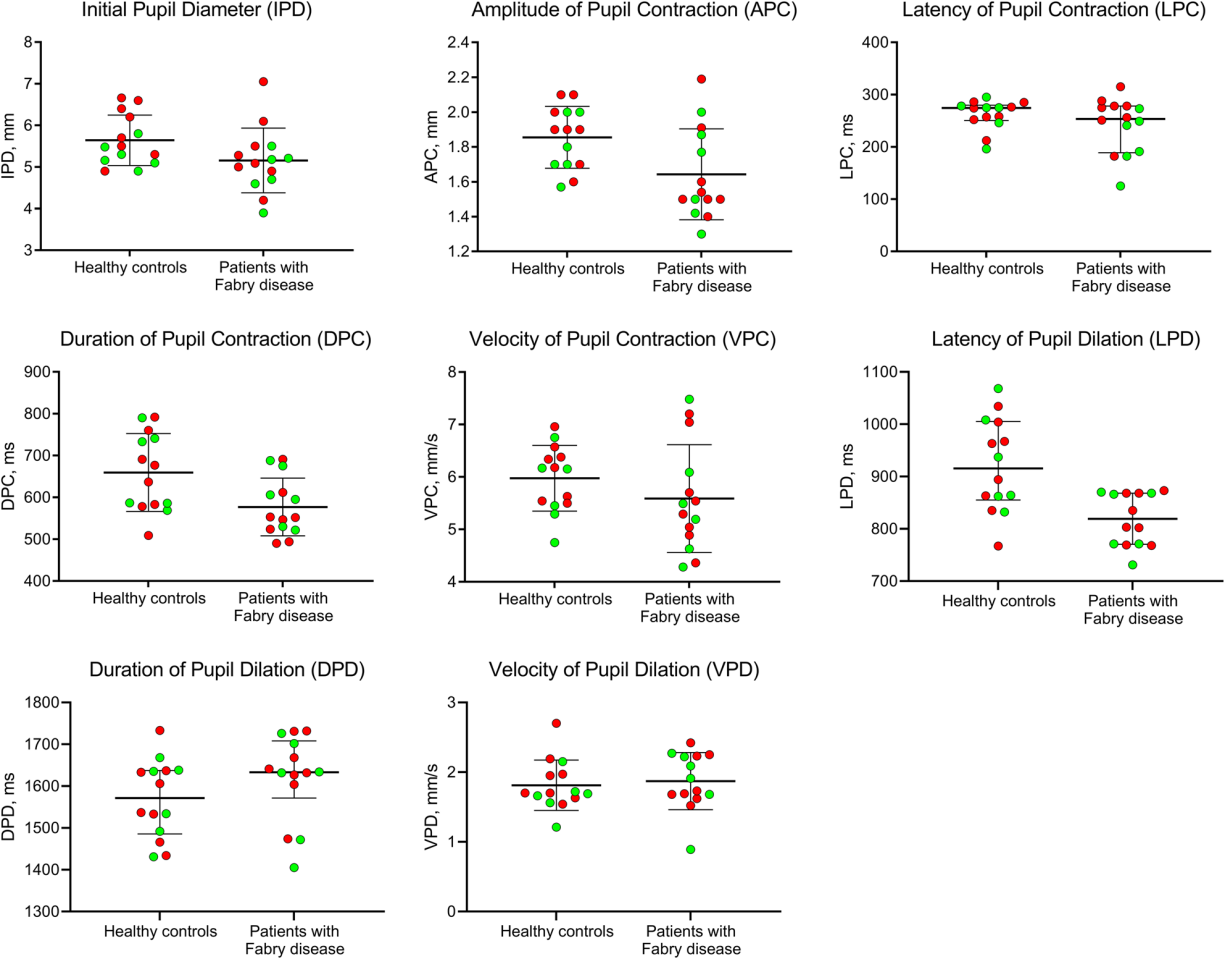
Dynamic pupillometry parameters in healthy control subjects and patients with Fabry disease, showing a significant reduction in APC (*P* = 0.048), DPC (*P* = 0.048) and LPD (*P* = 0.048), and no change in IPD (*P* = 0.154), LPC (*P* = 0.326), VPC (*P* = 0.326), DPD (*P* = 0.326), and VPD (*P* = 0.688) in Fabry patients. Red and green dots represent male and female participants, respectively.

There were significant reductions in the amplitude of pupil contraction (mean ± SD, 1.64 ± 0.26 vs. 1.86 ± 0.18 mm, *P* = 0.048), duration of pupil contraction (mean ± SD, 577.1 ± 69.0 vs. 659.5 ± 93.0 ms, *P* = 0.048), and latency of pupil dilation (median [IQR], 819.0 [770.5–868.0] vs. 915.5 [855.3–1005.0] ms, *P* = 0.048) in patients with Fabry disease compared to healthy control subjects. There was no significant difference in initial pupil diameter and other parameters (Table 2, Fig. 1). There were no significant differences in any of the pupil parameters between Fabry patients who were or were not being treated with ERT. No significant difference was observed in any of the pupil parameters between the right and left eyes of patients and controls (*P* > 0.05 for all).

**Table 2:** Pupillary light reflex responses in patients with Fabry disease and healthy control subjects.

	Controls (n = 14)	Fabry disease (n = 14)	*P* value	Adjusted *P* value^c^
Initial pupil diameter (mm)	5.64 ± 0.61	5.16 ± 0.77	0.077^a^	0.154
Amplitude of contraction (mm)	1.86 ± 0.18	1.64 ± 0.26	**0.018**^**a**^	**0.048**
Latency of contraction (ms)	274.5 (250.5–279.8)	253.5 (188.8–278.0)	0.285^b^	0.326
Duration of contraction (ms)	659.5 ± 93.0	577.1 ± 69.0	**0.013**^**a**^	**0.048**
Velocity of contraction (mm/s)	5.98 ± 0.63	5.59 ± 1.03	0.238^a^	0.326
Latency of dilation (ms)	915.5 (855.3–1005.0)	819.0 (770.5–868.0)	**0.012**^**b**^	**0.048**
Duration of dilation (ms)	1571.5 (1485.5–1637.3)	1633.0 (1571.5–1708.0)	0.265^b^	0.326
Velocity of dilation (mm/s)	1.81 ± 0.36	1.87 ± 0.41	0.688^a^	0.688

Data are expressed as mean ± SD for parametric variables and median (IQR) for non-parametric variables.

The bold *P* values represent statistically significant differences.

^a^Independent samples t-test.

^b^Mann Whitney U-test.

^c^*P* values were adjusted using a Benjamini–Hochberg false discovery rate (FDR) correction procedure.

The MSSI score (mean ± SD, 25.0 ± 8.9 vs. 15.0 ± 3.9, *P* = 0.045) was significantly higher in males compared to females with Fabry disease. Pupillary light reflex parameters and COMPASS 31 scores showed no difference between male and female patients with Fabry disease (Table 3). There were no significant differences in any of the pupil parameters between male and female controls.

**Table 3:** Comparison of the study parameters among male and female subjects with Fabry disease.

	Males with Fabry disease (n = 8)	Females with Fabry disease (n = 6)	*P* value	Adjusted *P* value^c^
Initial pupil diameter (mm)	5.39 ± 0.86	4.84 ± 0.57	0.208^a^	0.651
Amplitude of contraction (mm)	1.64 ± 0.27	1.64 ± 0.28	0.996^a^	0.996
Latency of contraction (ms)	276.5 (252.3–285.5)	216.0 (167.8–255.0)	**0.020**^**b**^	0.160
Duration of contraction (ms)	557.9 ± 66.2	602.7 ± 69.8	0.244^a^	0.651
Velocity of contraction (mm/s)	5.63 ± 1.00	5.53 ± 1.15	0.858^a^	0.981
Latency of dilation (ms)	819.0 (777.3–868.0)	818.5 (761.0–868.5)	0.852^b^	0.981
Duration of dilation (ms)	1636.5 (1609.8–1715.3)	1633.0 (1455.3–1708.0)	0.573^b^	0.981
Velocity of dilation (mm/s)	1.89 ± 0.35	1.84 ± 0.51	0.834^a^	0.981
Clinical characteristics				
α-Galactosidase A enzyme activity (µmol/L/h)	0.93 ± 0.52	2.25 ± 1.13	**< 0.001**^**a**^	**0.002**
Duration of ERT (months)	29.4 ± 9.3	15.0 ± 6.0	**0.023**^**a**^	**0.045**
MSSI	25.0 ± 8.9	15.0 ± 6.0	**0.027**^**a**^	**0.045**
COMPASS 31 Total weighted score	28.3 ± 14.9	28.8 ± 15.4	0.842^a^	0.956
COMPASS 31 pupillomotor weighted sub-score	2.2 ± 1.2	2.3 ± 0.7	0.956^a^	0.956

Data are expressed as mean ± SD for parametric variables and median (IQR) for non-parametric variables.

*COMPASS* Composite Autonomic Symptom Scale, *ERT* enzyme replacement therapy, *MSSI* Mainz Severity Score Index.

^a^Independent samples t-test.

^b^Mann Whitney U-test.

^c^*P* values were adjusted using a Benjamini–Hochberg false discovery rate (FDR) correction procedure.

The bold *P* values represent statistically significant differences.

### Correlations

**Figure 2:**
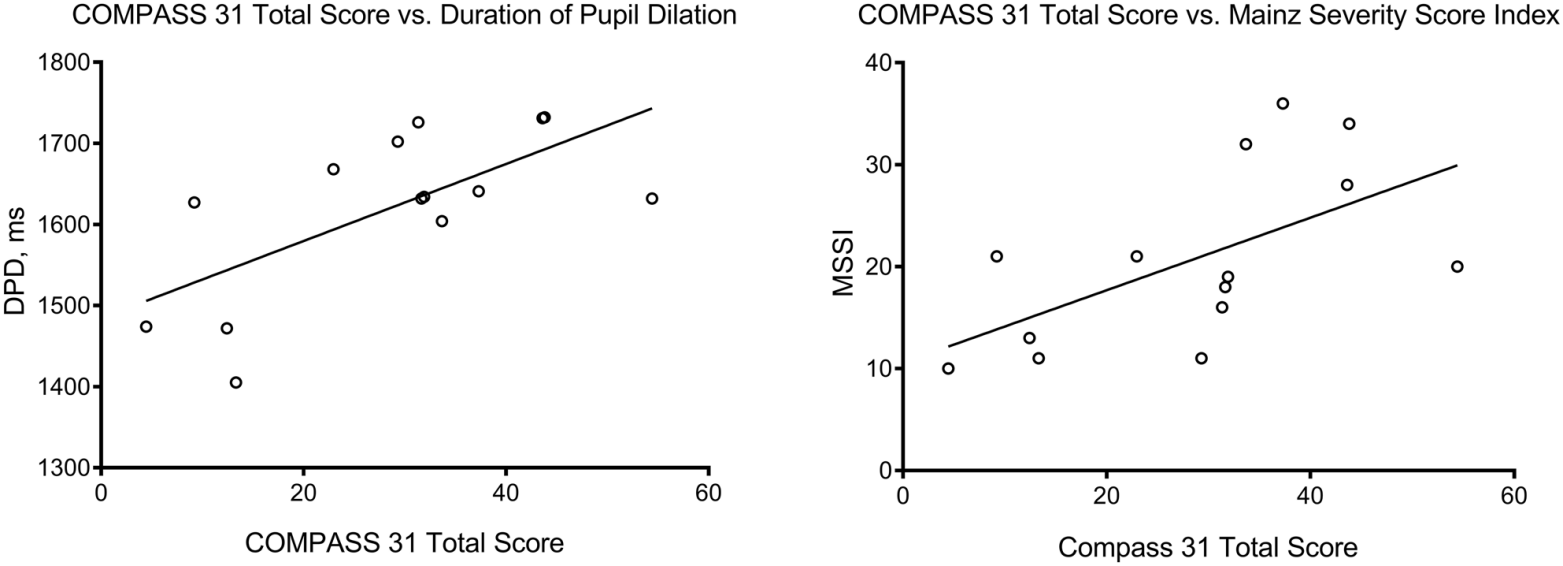
Scatter-plot graphs showing a significant correlation of COMPASS 31 total score with the duration of pupil dilation (ρ = 0.561; *P* = 0.037) and MSSI (*r* = 0.592; *P* = 0.026).

**Figure 3:**
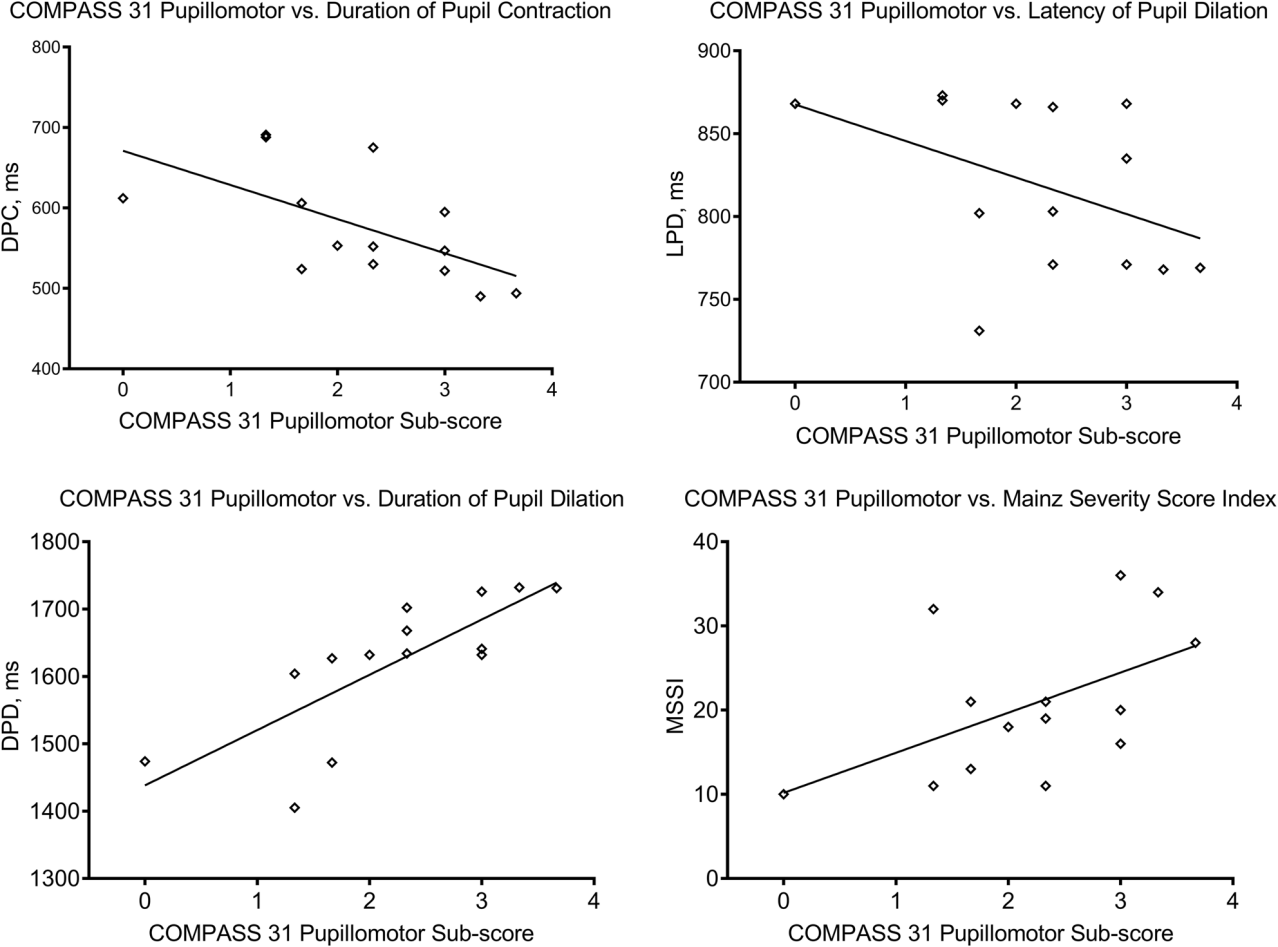
Scatter-plot graphs showing a significant correlation of the pupillomotor weighted sub-score of COMPASS 31 with the duration of pupil contraction (*r* = − 0.600; *P* = 0.023), latency of pupil dilation (ρ = − 0.541; *P* = 0.046), duration of pupil dilation (ρ = 0.877; *P* < 0.001), and MSSI (*r* = 0.533; *P* = 0.049).

The total weighted COMPASS 31 score showed a positive correlation with the duration of pupil dilation (ρ = 0.561; *P* = 0.037) and MSSI (*r* = 0.592; *P* = 0.026) (Fig. 2). The pupillomotor weighted COMPASS 31 sub-score showed an inverse correlation with the duration of pupil contraction (*r* = − 0.600; *P* = 0.023) and the latency of pupil dilation (ρ = − 0.541; *P* = 0.046), and a positive correlation with the duration of pupil dilation (ρ = 0.877; *P* < 0.001) and MSSI (*r* = 0.533; *P* = 0.049) (Fig. 3). The duration of ERT use correlated with MSSI (*r* = 0.797; *P* = 0.003), but there was no correlation with the pupillary parameters or COMPASS 31 scores. Age and MSSI did not correlate with pupil parameters.

## Discussion

This study shows that the amplitude and duration of pupil contraction and the latency of pupil dilation were reduced in patients with Fabry disease compared to controls. Furthermore, the alterations in pupillary light responses were related to the severity of autonomic symptoms assessed by the COMPASS 31 questionnaire. To the best of our knowledge, this is the first study reporting alterations in dynamic pupillary light responses in patients with Fabry disease.

Peripheral nerve involvement in Fabry disease is common but nerve conduction studies are often normal due to a predominant involvement of small and autonomic nerve fibers^19–21^. Previous studies using quantitative sensory testing have demonstrated small nerve fiber dysfunction in Fabry disease^22,23^. A reduction in small unmyelinated fibers has been demonstrated in sural nerve and skin biopsies^7,23,24^. We have previously used corneal confocal microscopy to show a loss of corneal nerve fibers and a relationship to disease severity in patients with Fabry disease^25,26^. There is also histopathological evidence of abnormal lipid deposition in central autonomic nuclei^6^ and degeneration of peripheral autonomic nerves^7^, which may contribute to autonomic dysfunction in Fabry disease. Indeed, conventional autonomic function tests including the sympathetic skin response, quantitative sudomotor axon reflex test and heart rate variability have been reported to be impaired in patients with Fabry disease^1,10–12^.

Quantitative evaluation of the pupillary light reflex utilizing dynamic pupillometry allows a rapid and objective assessment of sympathetic and parasympathetic nerve function. The initial pupil diameter i.e. the resting diameter in complete darkness, is mainly controlled by sympathetic activity, while the amplitude and velocity of pupil contraction indicate parasympathetic activity^27^. The latency of pupil dilation reflects the time from the end of the light stimulus to the beginning of pupil dilation and a longer latency indicates increased parasympathetic tone^17^. Therefore, the reduced amplitude and duration of pupillary contraction and reduced latency of pupil dilation indicate predominantly parasympathetic dysfunction in Fabry disease. A previous study of 45 patients with multiple sclerosis has also demonstrated a reduced amplitude of pupil contraction, indicative of parasympathetic dysfunction which was related to spinal cord atrophy^15^. Similarly, reduced amplitude and velocity, and increased latency of pupillary contraction have been observed in patients with Parkinson’s disease^16^. However, both studies reported no relationship between pupillometry parameters and measures of disease severity. We have also not observed an association between pupillary responses and MSSI, however there was a correlation between pupil parameters and the severity of autonomic symptoms as assessed by the COMPASS 31 questionnaire. More specifically, the pupillomotor weighted COMPASS 31 sub-score inversely correlated with the duration of pupil contraction and the latency of pupil dilation, indicating a relationship between pupillary parasympathetic dysfunction and the severity of pupillomotor symptoms. Previous studies have validated COMPASS 31 as an effective screening tool for the evaluation of autonomic neuropathy in Parkinson’s disease and diabetic autonomic neuropathy^28–30^. A recent study has shown abnormalities in thermal thresholds and reduced intraepidermal nerve fiber density, but no relationship with autonomic symptoms in patients with Fabry disease^31^. However, in the present study we found that both the total score and the pupillomotor weighted sub-score of COMPASS 31 correlate with an altered pupil light response and severity of Fabry disease assessed by the MSSI.

The level of α-galactosidase A enzyme activity was lower and the duration of ERT and the MSSI score were higher in males compared to females with Fabry disease, indicating more severe disease. Indeed, hemizygous males have more severe symptoms compared to heterozygous females^25,32,33^. In the present study whilst the MSSI score was higher in male compared to female patients with Fabry disease, there was no difference in the pupillary parameters and COMPASS 31 total and pupillomotor scores. The lack of significant difference in pupillary parameters between male and female patients despite the significant difference in disease severity may be attributed to the small sample size. In our earlier study we showed a greater MSSI score but comparable measures of neuropathic severity and corneal nerves using a first generation white light corneal confocal microscope when comparing male and female patients with Fabry disease^25^. However, more recently we showed a higher MSSI score and a significant reduction in both corneal nerve fiber density and length indicative of more severe disease in males compared to females with Fabry disease^26^.

Previous studies have demonstrated an improvement in Fabry-related cardiac autonomic dysfunction after ERT^34,35^. In our cohort of patients with Fabry disease, 79% were receiving ERT for a mean duration of 24 months. Despite treatment with ERT, there were significant impairments in pupillary light responses. Although the sample size of the subgroups was small, there was no difference in pupil parameters between patients with and without ERT use.

A limitation of this study is the small sample size, however, Fabry disease is a rare disorder. One might also argue that dynamic pupillometric evaluation should have been correlated with an objective measure of autonomic dysfunction, however, the COMPASS 31 score is a validated measure of the clinical severity of autonomic symptoms and therefore has more clinical relevance.

In conclusion, we show abnormal pupillary light responses which were related to the severity of autonomic symptoms in patients with Fabry disease. Further larger longitudinal studies are required to determine the utility of dynamic pupillometry for monitoring progression and outcomes in Fabry disease.

## Methods

Fourteen consecutive patients with Fabry disease and 14 age- and sex-matched healthy control subjects participated in this cross-sectional study undertaken at a single tertiary referral university hospital. The study was conducted in accordance with the principles of the Declaration of Helsinki and was approved by the Research Ethics Committee of the Necmettin Erbakan University. Written informed consent was obtained from all participants after a detailed explanation of the nature of the study.

The diagnosis of Fabry disease was based on clinical findings, evidence of a reduction of plasma α-galactosidase A enzyme activity and specific gene mutation analysis^36^. Exclusion criteria included previous ocular surgery or trauma, diabetes or any other neurological disorders that might cause autonomic dysfunction or use of topical or systemic medications that might influence autonomic function. Mainz Severity Score Index (MSSI) was used to assess the severity of Fabry disease and the Composite Autonomic Symptom Scale 31 (COMPASS 31) questionnaire was used to measure the severity of symptoms of autonomic dysfunction. The score for MSSI ranges from 0 to 76, including scores for general (0–18), neurological (0–20), renal (0–18) and cardiovascular (0–20) signs and symptoms of Fabry disease^37^. The COMPASS 31 questionnaire consists of 31 items in 6 domains (orthostatic intolerance, vasomotor, secretomotor, gastrointestinal, bladder, and pupillomotor). The total weighted scores of COMPASS 31 range from 0 to 100, and the pupillomotor weighted sub-scores range from 0 to 5, with a higher score indicating greater autonomic dysfunction^38^.

### Dynamic pupillary assessment

**Figure 4:**
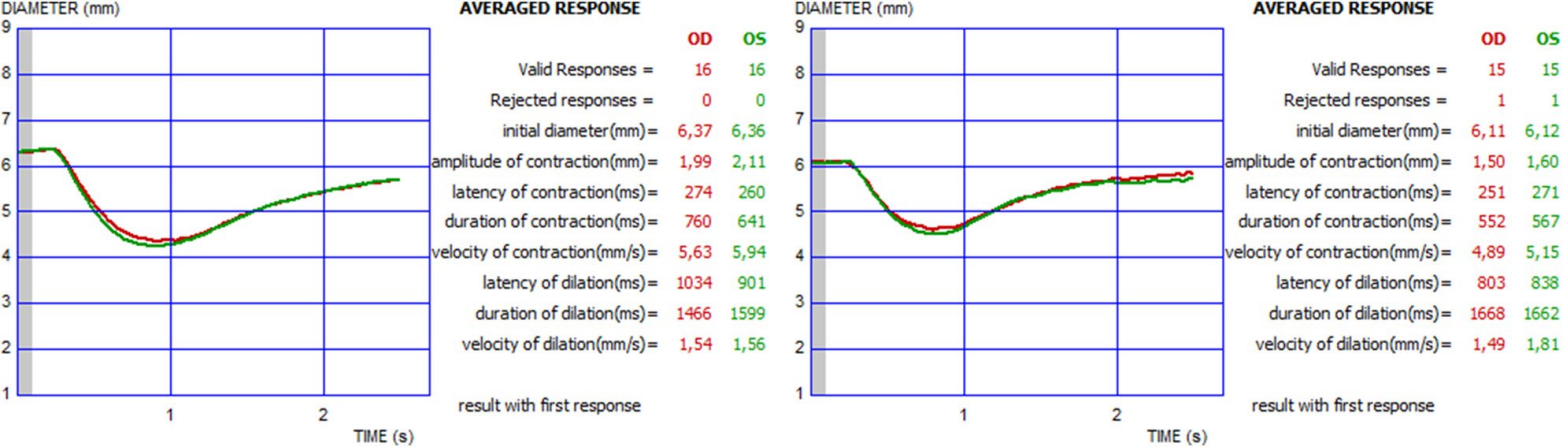
Binocular pupillary light reflex responses measured with dynamic pupillometry in a healthy subject (left), and a patient with Fabry disease (right).

Quantitative pupillary light reflex measurements were undertaken using an infrared dynamic pupillometry unit (MonPack One; Metrovision, France). This device is equipped with a near-infrared illumination source (880 nm) and a high-resolution infrared image sensor which records the pupil light responses in complete darkness. White light flashes (total luminance 100 cd/m^2^, stimulation on time 200 ms, off time 3300 ms) were used to elicit the pupillary light reflex, and automated real-time image processing was performed on the obtained images (30 images per second). The contours of the pupil were outlined by the proprietary software provided in the device, with a measurement sensitivity of 0.1 mm. The examinations were undertaken in a specially designed room with black walls and black, light-proof curtains. An additional light-proof curtain was used to separate the participant area in the room from the examiner to provide a completely dark environment, which also prevented accommodation. Binocular dynamic pupillary responses were recorded after 5 min of dark adaptation, considered to be an adequate time period for adaptation of cones for pupillary assessment^39^, and at least ten measurements were performed. The following eight parameters were automatically quantified: initial pupil diameter (mm), amplitude of pupil contraction (mm), latency of pupil contraction (ms), duration of pupil contraction (ms), velocity of pupil contraction (mm/s), latency of pupil dilation (ms), duration of pupil dilation (ms), and velocity of pupil dilation (mm/s) (Fig. 4). All pupillometric measurements were performed between 9 and 12 AM in order to reduce the potential effect of diurnal variation on the results. For all subjects, the data obtained from the right eyes were included in analyses.

### Statistical analysis

Statistical analysis of the data was performed with SPSS 21.0 (SPSS for Windows, USA) software. Basic descriptive statistics were calculated and reported as the mean ± SD or median (interquartile range [IQR]), as appropriate. Normal distribution of continuous variables was confirmed with the Kolmogorov–Smirnov test. The Pearson χ^2^ test was used to compare the categorical parameters. Independent samples t-test for normally distributed data and Mann Whitney U-test for non-normally distributed data were used to compare parameters between patients with Fabry disease and control participants. The Benjamini–Hochberg False Discovery Rate (FDR) correction procedure was applied to the *P* values to address the issue of multiple comparisons. The associations between disease severity scores, autonomic symptom scores and pupillary light reflex parameters were measured using Pearson’s correlation coefficient for parametric data and Spearman’s correlation coefficient for non-parametric data. For all evaluations, a *P* value of less than 0.05 was considered statistically significant.

## Data Availability

The datasets generated during and/or analyzed during the current study are available from the corresponding author on reasonable request.

## References

[1] Hilz MJ (2002). Evaluation of peripheral and autonomic nerve function in Fabry disease. Acta Paediatr. Suppl..

[2] Zarate YA, Hopkin RJ (2008). Fabry's disease. Lancet.

[3] Kahn P (1973). Anderson-Fabry disease: A histopathological study of three cases with observations on the mechanism of production of pain. J. Neurol. Neurosurg. Psychiatry..

[4] Rahman AN, Lindenberg R (1963). The neuropathology of hereditary dystopic lipidosis. Arch. Neurol..

[5] Tabira T, Goto I, Kuroiwa Y, Kikuchi M (1974). Neuropathological and biochemical studies in Fabry's disease. Acta Neuropathol..

[6] Kaye EM, Kolodny EH, Logigian EL, Ullman MD (1988). Nervous system involvement in Fabry's disease: Clinicopathological and biochemical correlation. Ann. Neurol..

[7] Liguori R (2010). Small fiber neuropathy in female patients with Fabry disease. Muscle Nerve..

[8] Cable WJ, Kolodny EH, Adams RD (1982). Fabry disease: Impaired autonomic function. Neurology.

[9] Kolodny EH, Pastores GM (2002). Anderson-Fabry disease: Extrarenal, neurologic manifestations. J. Am. Soc. Nephrol..

[10] Biegstraaten M, van Schaik IN, Wieling W, Wijburg FA, Hollak CE (2010). Autonomic neuropathy in Fabry disease: A prospective study using the Autonomic Symptom Profile and cardiovascular autonomic function tests. BMC. Neurol..

[11] Gomes I (2003). Nerve conduction studies, electromyography and sympathetic skin response in Fabry’s disease. J. Neurol. Sci..

[12] Kampmann C (2008). Cardiac manifestations of Anderson-Fabry disease in children and adolescents. Acta Paediatr..

[13] Lerner AG (2015). Type 2 diabetes and cardiac autonomic neuropathy screening using dynamic pupillometry. Diabet Med..

[14] Ferrari GL (2010). Using dynamic pupillometry as a simple screening tool to detect autonomic neuropathy in patients with diabetes: A pilot study. Biomed. Eng. Online..

[15] de Seze J (2001). Pupillary disturbances in multiple sclerosis: Correlation with MRI findings. J. Neurol. Sci..

[16] Giza E, Fotiou D, Bostantjopoulou S, Katsarou Z, Karlovasitou A (2011). Pupil light reflex in Parkinson's disease: evaluation with pupillometry. Int. J. Neurosci..

[17] Aydogmus Y (2017). Is overactive bladder a nervous or bladder disorder? Autonomic imaging in patients with overactive bladder via dynamic pupillometry. World J. Urol..

[18] Chougule PS, Najjar RP, Finkelstein MT, Kandiah N, Milea D (2019). Light-induced pupillary responses in Alzheimer's disease. Front. Neurol..

[19] Burlina AP (2011). Early diagnosis of peripheral nervous system involvement in Fabry disease and treatment of neuropathic pain: The report of an expert panel. BMC Neurol..

[20] Akpinar ÇK, Türker H, Bayrak O, Cengiz N (2015). Electroneuromyographic features in Fabry disease: A retrospective review. Noro. Psikiyatr. Ars..

[21] Franques J (2017). Peripheral nerve involvement in Fabry's disease: Which investigations? A case series and review of the literature. Rev. Neurol. (Paris).

[22] Luciano CA (2002). Physiological characterization of neuropathy in Fabry’s disease. Muscle Nerve..

[23] Üçeyler N (2011). Small fibers in Fabry disease: Baseline and follow-up data under enzyme replacement therapy. J. Peripher. Nerv. Syst..

[24] Toyooka K, Said G (1997). Nerve biopsy findings in hemizygous and heterozygous patients with Fabry’s disease. J. Neurol..

[25] Tavakoli M (2009). Corneal confocal microscopy: A novel noninvasive means to diagnose neuropathy in patients with Fabry disease. Muscle Nerve..

[26] Bitirgen G, Turkmen K, Malik RA, Ozkagnici A, Zengin N (2018). Corneal confocal microscopy detects corneal nerve damage and increased dendritic cells in Fabry disease. Sci. Rep..

[27] Yamaji K, Hirata Y, Usui S (2000). A method for monitoring autonomic nervous activity by pupillary flash response. Syst. Comp. Jpn..

[28] Kim Y (2017). The composite autonomic symptom scale 31 is a useful screening tool for patients with Parkinsonism. PLoS ONE.

[29] Greco C (2017). Validation of the Composite Autonomic Symptom Score 31 (COMPASS 31) for the assessment of symptoms of autonomic neuropathy in people with diabetes. Diabet. Med..

[30] Singh R, Arbaz M, Rai NK, Joshi R (2019). Diagnostic accuracy of composite autonomic symptom scale 31 (COMPASS-31) in early detection of autonomic dysfunction in type 2 diabetes mellitus. Diabetes Metab. Syndr. Obes..

[31] Siedler G (2019). Dyshidrosis is associated with reduced amplitudes in electrically evoked pain-related potentials in women with Fabry disease. Clin. Neurophysiol..

[32] Ries M (2003). The early clinical phenotype of Fabry disease: A study on 35 European children and adolescents. Eur. J. Pediatr..

[33] Turkmen K, Baloglu I (2020). Fabry disease: Where are we now?. Int. Urol. Nephrol..

[34] Ries M (2006). Enzyme-replacement therapy with agalsidase alfa in children with Fabry disease. Pediatrics.

[35] Hilz MJ (2010). Enzyme replacement therapy improves cardiovascular responses to orthostatic challenge in Fabry patients. J. Hypertens..

[36] Turkmen K (2016). The prevalence of Fabry disease in patients with chronic kidney disease in Turkey: The TURKFAB study. Kidney Blood Press. Res..

[37] Beck M (2006). The Mainz Severity Score Index (MSSI): Development and validation of a system for scoring the signs and symptoms of Fabry disease. Acta Paediatr. Suppl..

[38] Sletten DM, Suarez GA, Low PA, Mandrekar J, Singer W (2012). COMPASS 31: A refined and abbreviated Composite Autonomic Symptom Score. Mayo Clin. Proc..

[39] Flanagan SC, Saunders KJ, Queener HM, Richardson P, Ostrin LA (2020). Effects of mydriatics on rod/cone- and melanopsin-driven pupil responses. Optom. Vis. Sci..

